# Reliability of Gait Analysis Using ORPHE ANALYTICS in Patients with Type 2 Diabetes: A Single-center Observational Study

**DOI:** 10.31662/jmaj.2024-0422

**Published:** 2025-06-13

**Authors:** Takaaki Matsuda, Yoshinori Osaki, Hirofumi Takahashi, Erika Matsuda, Yasuhiro Suzuki, Kosuke Kojo, Yuki Murayama, Yoko Sugano, Hitoshi Iwasaki, Bryan J. Mathis, Hiroaki Suzuki, Motohiro Sekiya, Hitoshi Shimano

**Affiliations:** 1 Department of Endocrinology and Metabolism, University of Tsukuba Hospital, Tsukuba, Japan; 2 Tsukuba Clinical Research & Development Organization (T-CReDO), University of Tsukuba, Tsukuba, Japan; 3 Department of Endocrinology and Metabolism, Institute of Medicine, University of Tsukuba, Tsukuba, Japan; 4 Nutrition Department, University of Tsukuba Hospital, Tsukuba, Japan; 5 Institute of Systems and Information Engineering, University of Tsukuba, Tsukuba, Japan; 6 Department of Urology, University of Tsukuba Hospital, Tsukuba, Japan; 7 Department of Cardiovascular Surgery, Institute of Medicine, University of Tsukuba, Tsukuba, Japan; 8 Department of Food and Health Sciences, Faculty of Human Life Sciences, Jissen Women’s University, Tokyo, Japan

**Keywords:** gait analysis, type 2 diabetes, elderly, reliability, inertial measurement unit, wearable devices

## Abstract

**Introduction:**

People with diabetes tend to show abnormalities in gait parameters, including walking speed and stride length, relative to those without diabetes. While inertial measurement units (IMUs) provide a portable alternative to optical motion capture systems, the reliability of gait analysis is influenced by factors such as walking distance, timing, and examiner differences. However, the impact of these parameters on gait analysis in patients with type 2 diabetes (T2D) remains unclear. This study aimed to evaluate the reliability of ORPHE ANALYTICS, an IMU-based gait analysis system, under varying measurement conditions in patients with T2D.

**Methods:**

We conducted a single-center observational study (n = 9) to clarify the reliability of ORPHE ANALYTICS, a gait analysis motion sensor system developed by ORPHE Inc., which evaluates more than 15 gait parameters, in patients with T2D. The relative reliability was assessed using intraclass correlation coefficients (ICC): ICC_(1,1)_ or ICC_(1,3)_ for intra-rater reliability and ICC_(2,1)_ for inter-rater reliability based on the differences in distance (10 vs. 30 m), examiners, and timing (morning vs. afternoon).

**Results:**

Intra-rater reliability was excellent (ICC_(1,1)_ and ICC_(1,3)_ ≥0.9) for all gait parameters except coefficient of variation of stride duration and lateral displacement. Measurements taken under different conditions of distance and timing exhibited almost good inter-rater reliability (ICC_(2,1)_ ≥0.75), while measurements by different examiners exhibited moderate to good reliability (ICC_(2,1)_ ≥0.50). Significant novel differences were observed in lateral sway during the swing phase, medial sway during the stance phase, and foot angle, with random errors (expressed as percentage of minimal detectable change) exceeding 40% under various measurement conditions.

**Conclusions:**

ORPHE ANALYTICS exhibited good to excellent intra-rater and inter-rater reliability based on differences in distance and timing. However, persistent inter-rater reliability challenges in patients with T2D warrant analysis by a single examiner.

## Introduction

A relationship between gait abnormalities and disease has been reported not only in neurodegenerative disorders such as dementia ^(1), (2), (3)^, Huntington’s disease ^(4)^, multiple sclerosis ^(4)^, and Parkinson’s disease ^(5)^, but also in diabetes. Studies comparing individuals with and without diabetes have observed differences in walking speed, stride length, walking time, stance time, and joint range of motion ^(6), (7)^. Furthermore, among individuals with diabetes, the presence or absence of neuropathy has been reported to influence these gait parameters ^(6), (7), (8)^. To evaluate these parameters, most reports use an optical motion capture system, which is the gold standard for gait analysis. Recently, gait analysis using an inertial measurement unit (IMU), which is easy to use and portable, has become more popular. IMU-based gait analyses have shown differences in walking speed, landing angle, and maximum swing angle between people with and without diabetes, clarifying how peripheral neuropathy affects these gait parameters ^(9), (10)^.

However, gait analysis is also affected by test conditions, namely the timing of the test ^(11)^, the gait distance ^(12), (13)^, and various other factors. A meta-analysis also reported that healthy individuals walk faster in the evening ^(11)^, whereas patients with Alzheimer’s disease perform better in the morning ^(14)^ and those with Parkinson’s disease show improved gait in the afternoon ^(15)^. Additionally, longer distances improve reliability and reduce variability ^(13)^, but constrained clinical environments may render this challenging. Therefore, when applying IMUs to clinical practice, an understanding of how testing conditions influence measurements of gait parameters is required. However, the impact of these factors on gait analysis in those with type 2 diabetes (T2D) and their usefulness in measuring spatiotemporal parameters remain unclarified. Furthermore, the reliability of various gait analysis devices has not been fully investigated.

ORPHE ANALYTICS is a validated IMU embedded in shoe midsoles for gait analysis, developed by ORPHE Inc., and has demonstrated high concurrent validity compared with optical motion capture systems ^(16)^. This study aimed to evaluate the intra-rater reliability of ORPHE ANALYTICS and to investigate whether differences in walking distance, timing, and examiner affect the reliability of the gait analysis system in patients with T2D. The findings aim to provide foundational data for future clinical applications.

## Materials and Methods

### Study design and setting

This study was a single-center observational study of individuals with T2D who were admitted to the Department of Endocrinology and Metabolism at the University of Tsukuba Hospital from September 1 to November 30, 2023.

### Participants

We included in the study patients aged >40 years with T2D who did not meet the exclusion criteria and agreed to participate in the study. The exclusion criteria were as follows: i) diabetes other than T2D; ii) a history of or comorbidity with central nervous system diseases, a history of head injury, or psychiatric disorders affecting cognitive function; iii) difficulties in normal walking due to orthopedic disorders or the use of walking support devices; iv) visual or hearing impairments; v) difficulty communicating in Japanese; vi) pregnancy; vii) newly diagnosed diabetes or hyperglycemic emergency; and viii) any determination of ineligibility by the principal investigator.

### Variables and data collection

We collected the following information as baseline data: duration of diabetes, diabetic complications (neuropathy, retinopathy, nephropathy, and cardiovascular disease), smoking history, height, weight, body mass index (BMI), urinary albumin, hemoglobin A1C (HbA1c), glycoalbumin, creatinine, estimated glomerular filtration rate (eGFR), serum albumin, and creatine kinase.

Diabetic neuropathy was defined as two or more of the following three points: i) subjective symptoms thought to be caused by diabetic neuropathy, ii) decreased or absent bilateral Achilles tendon reflexes, and/or iii) decreased bilateral inner-malleolus vibration sense as indicated by a C128 tuning fork response of ≤10 seconds. Diabetic retinopathy was defined as the presence of more than simple retinopathy. Cardiovascular disease was defined as the presence of any of the following: cerebral infarction, ischemic heart disease, or peripheral arterial disease.

BMI was calculated by dividing body weight (kg) by the square of height (m), while eGFR was calculated on the basis of the Japanese eGFR estimation equation ^(17)^.

### Gait analysis

The participants wore shoes (SHIBUYA 2.0, ORPHE Inc., Tokyo, Japan) with motion sensors (ORPHE CORE, ORPHE Inc.) built into the midsoles for gait analysis (ORPHE ANALYTICS, ORPHE Inc.). The motion sensors were attached to both shoes, weighed approximately 20 g, measured 45 × 29 × 14 mm in size, and had a sampling frequency of 200 Hz. Details of the shoes and motion sensors were previously described ^(16)^. We prepared shoes in sizes ranging from 24 to 28 cm (Japanese Industrial Standards) at 0.5-cm increments. Participants selected appropriate shoes before testing and researchers ensured proper fit. Two examiners (examiners A and B) received training on the measurement method from ORPHE technical staff before the start of the study and subsequently conducted the measurements after using the device themselves.

**Figure 1.**
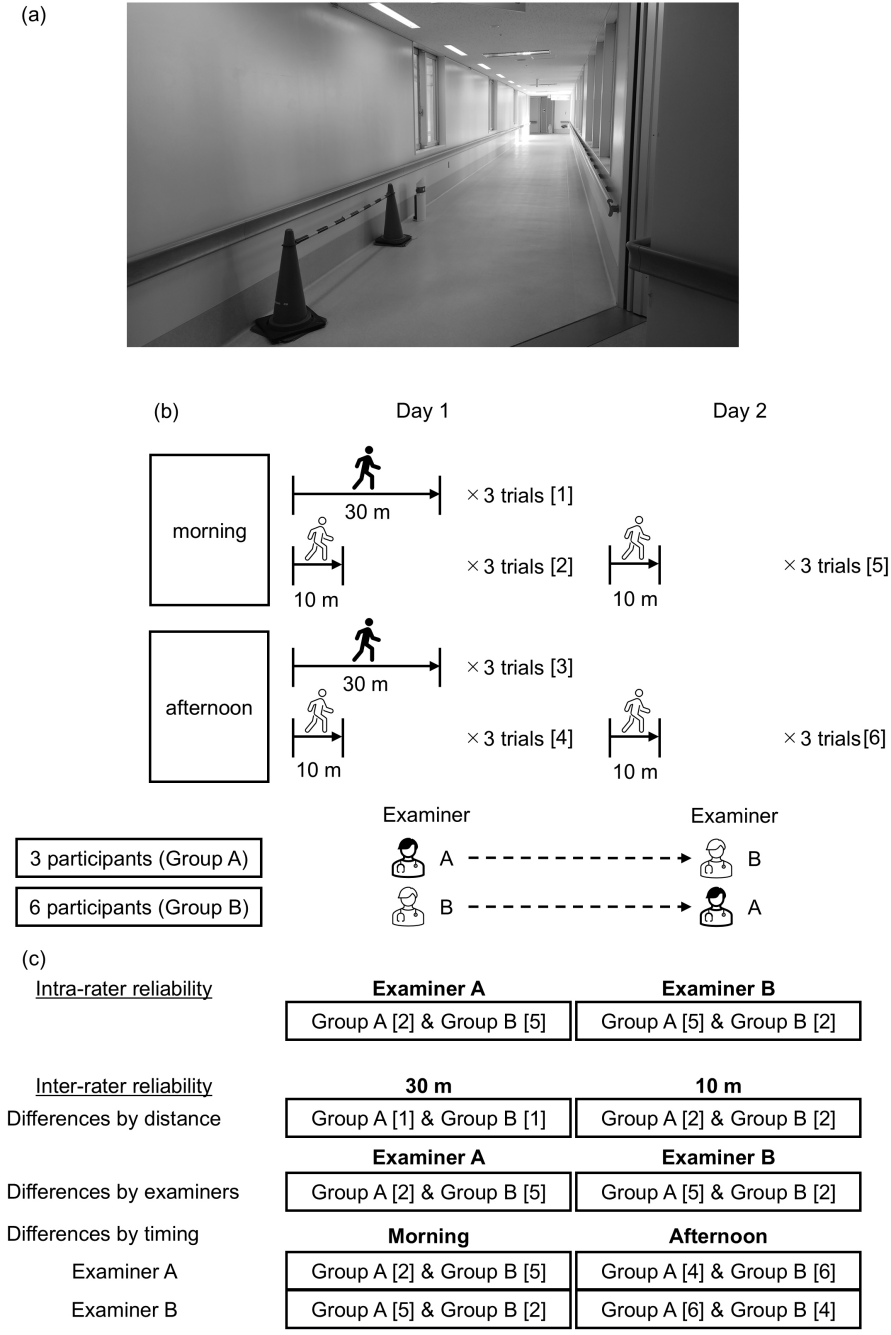
Gait analysis settings. The gait analysis was conducted in a long over 30-m-long corridor (a). The participants were tested under different conditions by distance (10 vs. 30 m), examiner (examiner A vs. B) and timing (morning vs. afternoon). Each condition was tested three times. Square brackets indicate trials based on distance and time for each measurement day. On day 1, examiner A measured three participants, while examiner B measured six participants. The participants measured by examiner A on day 1 were defined as group A, and those measured by examiner B were defined as group B (b). The measurement points used to evaluate intra-examiner and inter-examiner reliability based on distance, examiner, and timing are shown in (c). The difference in distance was analyzed using data from examiners A and B collected in the morning of day 1.

The gait analysis was conducted in an over 30-meter-long corridor in the University of Tsukuba Hospital, separated by traffic cones, with no other pedestrians present (Figure 1). Participants walked under varying conditions, including distance (10 vs. 30 m), timing (morning [8 AM-12 PM] vs. afternoon [12 PM-4 PM]), and examiner (A vs. B). We chose two different distances based on previous reports evaluating the reliability of IMU-based gait analyses ^(12), (18)^. Each condition was tested in three trials (Figure 1). The second gait analysis was conducted on the day after the first (not necessarily on consecutive days), and examiners were alternated. Participants walked at a comfortable pace, starting from a standing position. Examiners walked behind the participants to monitor the motion sensors without affecting walking speed. We did not set the acceleration/deceleration sections because the gait indices are calculated using the measurement values from the entire walking session with only interquartile range values included.

The measured gait parameters included speed, cadence, stride duration, stride length, coefficient of variation (CV) of stride duration (stride CV), foot angle, stance phase duration, swing phase duration, strike angle, toe-off angle, pronation, vertical height, landing impact, lateral maximum displacement, and lateral minimum displacement. These gait parameters were calculated as the average of the values measured for the left and right feet. The definitions of speed and vertical height were reported in previous studies ^(16)^, whereas other gait parameters are described in the table captions.

### Ethical approval

This study, based on the principles of the Declaration of Helsinki, was conducted with the approval of the Ethics Committee of the University of Tsukuba Hospital (approval date: July 27, 2023; approval number: R05-058). Written, informed consent was obtained from all participants.

### Statistical analysis

We performed the Shapiro-Wilk test using the baseline data expressed as a continuous variable, and present the results as the mean (with standard deviation [SD]) and median (with interquartile range) according to the distribution. Categorical variables are presented as n (%). We also conducted a Shapiro-Wilk test using the gait parameter values for a 10-m walk on the morning of day 1 and confirmed a normal distribution. We performed statistical analyses using IBM SPSS Statistics, Version 29.0 (IBM Corp., Armonk, NY, USA). A p-value of <0.05 was considered statistically significant.

#### Relative reliability

The relative reliability was evaluated using the intraclass correlation coefficient (ICC) with 95% confidence interval (CI). ICC values <0.5, 0.5-0.75, 0.75-0.90, and >0.90 were considered to indicate poor, moderate, good, and excellent reliability, respectively ^(19)^. To evaluate the intra-rater reliability for each examiner (A and B), we calculated ICC_(1,1)_ and ICC_(1,3)_ using the mean of the three trials. ICC_(1,1)_ was calculated using a one-way random effects model for a single rater with absolute agreement, while ICC_(1,3)_ used the same model but for multiple trials. To evaluate the inter-rater reliability for different distances (10 vs. 30 m), examiners (examiner A vs. B), and timing (morning vs. afternoon), we calculated ICC_(2,1)_. ICC_(2,1)_ was calculated using a two-way random effects model for a single rater with absolute agreement.

#### Absolute reliability

Bland-Altman analysis was performed to evaluate absolute reliability. The horizontal axis represented the mean value of the two measurements, and the vertical axis showed their difference. The Bland-Altman plot shows the mean (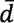) and 95% CI for the difference (*d*) between the two measurements in the sample population on the Y-axis, with the dotted line. These were calculated using the following formula (1), based on the SD (*SD*_d_) for *d* and the *z*-value (1.96) for the 95% CI.


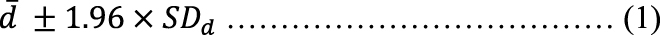


To investigate fixed bias as a form of systematic bias, we calculated the 95% CI for the mean difference between the two measurements (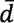) using the following formula (2), based on the sample size (*n*), the SD of the difference between the two measurements (*SD*_*d*_), and the t-value for (*n*−1) degrees of freedom ^(20)^.


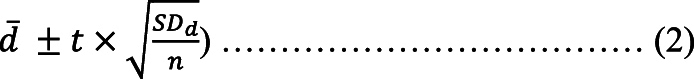


If the 95% CI included zero, no fixed bias was assumed. Proportional bias was assessed through linear regression analysis of the mean and difference ^(20)^. A significant result indicated proportional bias. The presence of either fixed or proportional bias was interpreted as evidence of systematic bias. The lower and upper coefficient limits of the agreement (LOA) in the sample population were calculated as follows (lower coefficient limit [LCL] (3) and upper coefficient limit [UCL] (4)).


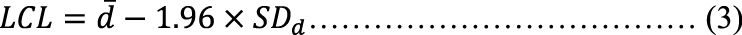



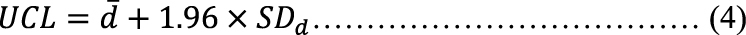


Furthermore, since equations (3) and (4) represent the UCL and LCL of the LOA in the sample population, the 95% CIs of the LCL and UCL were calculated using equations (5) and (6) to estimate the LOA in the population and are presented in Supplementary Table 1 ^(21)^. We considered the range between the upper limit of (5) and the lower limit of (6) to be the narrowest (optimistic) LOA of the estimated population.


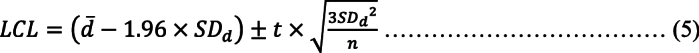



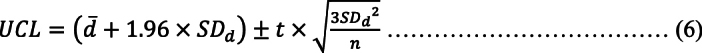


#### Random error

The standard error of measurement (SEM) (5) and minimal detectable change (MDC) (6) with 95% CI were calculated as follows ^(22)^:


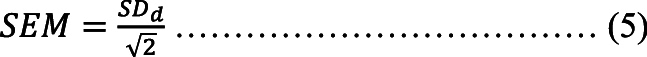



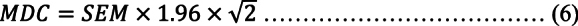


Percentages of SEM (%SEM) and MDC (%MDC) relative to the mean of the two measurements were also calculated. On the basis of previous studies ^(18)^, %SEM was classified as low (%SEM ≤10%) or high (%SEM >10%), while %MDC was categorized as low (%MDC ≤20%), acceptable (20% < %MDC < 40%), or high (%MDC ≥40%).

## Results

### Study participant flow

**Figure 2.**
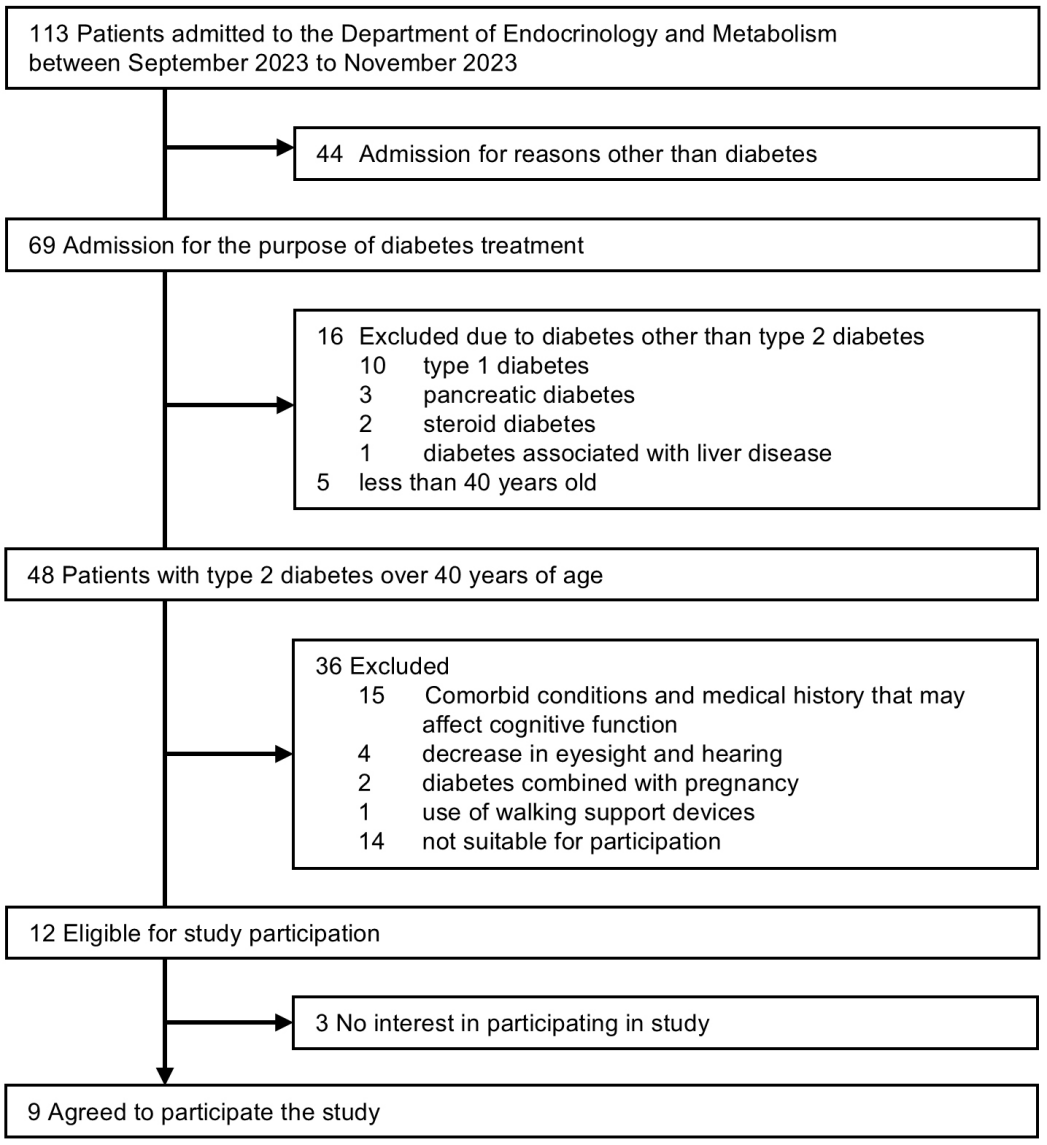
Flowchart of study participants. During the study period, 48 patients with diabetes were admitted to the Department of Endocrinology and Metabolism. Finally, 9 patients agreed to participate in this study.

A total of 48 patients with T2D aged 40 years or older were admitted to the Department of Endocrinology and Metabolism between September 1 and November 30, 2023, but 36 patients were excluded. Of the 12 patients meeting the inclusion criteria, 3 declined participation. Finally, 9 patients agreed to participate. Reasons for non-participation were painful blisters of pemphigus on their feet (n = 1), refusal to take a cognitive function test (n = 1), and no particular reason (n = 1) (Figure 2).

### Baseline characteristics of study participants

The mean age of the participants in this study was 68 (7) years; 44.4% were female, and mean disease duration was 17 (9) years (Table 1). The mean BMI was 23.7 (5.3) kg/m^2^ and the mean HbA1c value was 9.0% (1.3%). Of all participants, 88.9% had diabetic neuropathy, 66.7% had diabetic retinopathy, and 44.4% had overt albuminuria. Two participants had a history of cardiovascular disease (cerebral infarction [n = 1] and peripheral arterial disease [n = 1]), but we confirmed that they had no walking difficulties. The mean values of the gait parameters for the three trials for different conditions (distance, examiner, and timing) are shown in Table 2.

**Table 1.** Baseline Characteristics of Study Participants.

Variables	n = 9
Age (years)	68 (7)
Female, n (%)	4 (44.4)
Duration of diabetes (y)	17 (9)
Smoking, n (%)	7 (78)
Alcohol consumption	6 (66.7)
Body mass index (kg/m^2^)	23.7 (5.3)
HbA1c (%)	9.0 (1.3)
HbA1c (mmol/mol)	75 (14)
Glycated albumin (%)	22.8 (18.8-29.6)
Urinary albumin (mg/24 hours)	29 (4-50)
eGFR (mL/min/1.73 m^2^)	50 (21)
Albumin (g/dL)	4.2 (0.4)
Creatine kinase (U/L)	86 (41)
Hypertension, n (%)	5 (55.6)
Dyslipidemia, n (%)	7 (77.8)
Diabetic complications	
Neuropathy, n (%)	8 (88.9)
Retinopathy, n (%)	6 (66.7)
Nephropathy, n (%)	
Urinary albumin <30 mg/24 hours, n (%)	5 (55.6)
Urinary albumin 30-299 mg/24 hours, n (%)	3 (33.3)
Urinary albumin ≥300 mg/24 hours, n (%)	1 (11.1)
Cardiovascular disease, n (%)	2 (22.2)
Cerebral infarction, n (%)	1 (11.1)
Ischemic heart disease, n (%)	0 (0.0)
Peripheral arterial disease, n (%)	1 (11.1)

Data are expressed as means (SD), median (interquartile range), and n (%). eGFR: estimated glomerular filtration rate; HbA1c: hemoglobin A1C.

**Table 2.** Gait Parameters for Each Setting of the Study Participants.

		Length	10 m		10 m		30 m	
		timing	Morning	Afternoon	Morning	Afternoon	Morning	Afternoon
		examiner	A	A	B	B	A or B	A or B
Speed	m/sec		1.19 (0.14)	1.16 (0.15)	1.15 (0.14)	1.17 (0.15)	1.19 (0.14)	1.18 (0.16)
Cadence	steps/min		58 (4)	57 (3)	57 (3)	57 (4)	58 (3)	58 (4)
Stride duration	sec		1.04 (0.06)	1.05 (0.06)	1.06 (0.07)	1.06 (0.07)	1.04 (0.05)	1.04 (0.07)
Stride length	m		1.24 (0.15)	1.21 (0.16)	1.21 (0.14)	1.24 (0.15)	1.23 (0.15)	1.23 (0.16)
Stride CV	%		2.81 (0.61)	3.02 (0.80)	2.85 (0.71)	3.12 (1.04)	2.70 (0.47)	3.20 (1.86)
Foot angle	degree		-16.0 (4.4)	-15.5 (5.0)	-16.0 (9.5)	-17.2 (3.6)	-15.3 (9.3)	-17.1 (3.6)
Stance phase duration	sec		0.68 (0.04)	0.69 (0.04)	0.69 (0.04)	0.70 (0.04)	0.68 (0.03)	0.68 (0.04)
Swing phase duration	sec		0.36 (0.03)	0.37 (0.04)	0.37 (0.04)	0.37 (0.04)	0.36 (0.03)	0.36 (0.04)
%Stance phase duration	%		65 (2)	65 (2)	66 (2)	65 (2)	65 (2)	65 (2)
%Swing phase duration	%		35 (2)	35 (2)	34 (2)	35 (2)	35 (2)	35 (2)
Strike angle	degree		-17.2 (4.2)	-17.0 (4.0)	-15.8 (3.2)	-16.9 (3.4)	-16.2 (3.8)	-16.6 (3.6)
Toe-off angle	degree		-57 (6)	-55 (6)	-55 (5)	-56 (5)	-57 (6)	-57 (6)
Landing impact	m/sec^2^		65 (13)	63 (15)	65 (16)	66 (15)	68 (16)	67 (17)
Pronation	degree		-9.2 (2.5)	-9.0 (2.7)	-8.6 (5.4)	-9.8 (2.6)	-7.9 (5.4)	-9.4 (2.3)
Lateral maximum displacement	cm		1.72 (0.73)	1.72 (0.67)	1.79 (0.73)	2.04 (0.65)	1.75 (0.67)	1.88 (0.58)
Lateral minimum displacement	cm		-0.87 (0.36)	-0.83 (0.26)	-0.82 (0.31)	-0.66 (0.24)	-0.86 (0.40)	-0.74 (0.20)
Vertical height	cm		16.8 (1.1)	16.6 (1.2)	16.5 (1.1)	16.7 (1.3)	17.0 (1.3)	16.8 (1.3)

The foot angle represents the angle of the foot’s long axis relative to the stride direction (negative values indicate outward toe rotation); pronation represents the angle of inward foot roll upon landing (negative values indicate greater inward roll); the strike and toe-off angles represent the shoe-ground angles during landing and the foot lift-off (respectively) (negative values indicate higher toe/heel lift); landing impact was the peak acceleration (m/sec^2^) along the Z-axis at landing, and lateral maximum (minimum) displacement (cm) represents the maximum/minimum lateral foot deviation during the swing phase (positive values indicate outward, negative inward). The ratio of stance phase duration and swing phase duration in one gait cycle was expressed as %stance phase duration (%) and %swing phase duration (%). Data are expressed as means (SD). CV: coefficient of variation; SD: standard deviation.

### Intra-rater reliability

Table 3 shows the intra-rater reliability for three 10-m trials conducted by examiners A and B. Except for stride CV and lateral maximum (minimum) displacement, the ICC_(1,1)_ was 0.9 or higher for both examiners, indicating excellent relative intra-rater reliability, and suggesting that a single measurement would be sufficient. However, the stride CV showed low reliability, with ICC_(1,3)_ ranging from 0.3 to 0.5. The lateral maximum (minimum) displacement had good reliability, with ICC_(1,3)_ of 0.8 or more.

**Table 3.** Intra-Rater Reliability of the Motion Sensor-Based Gait Analysis System as Evaluated by Each of the Two Examiners.

	Examiner A				Examiner B			
	ICC_(1,1)_	95% CI	ICC_(1,3)_	95% CI	ICC_(1,1)_	95% CI	ICC_(1,3)_	95% CI
Speed	0.975	0.923-0.994	0.992	0.973-0.998	0.963	0.887-0.992	0.987	0.959-0.997
Cadence	0.931	0.797-0.984	0.976	0.922-0.995	0.965	0.893-0.992	0.988	0.962-0.997
Stride duration	0.911	0.747-0.979	0.968	0.898-0.993	0.960	0.878-0.991	0.986	0.956-0.997
Stride length	0.974	0.920-0.994	0.991	0.972-0.998	0.975	0.921-0.994	0.991	0.972-0.998
Stride CV	0.141	−0.218 to 0.659	0.331	−1.155 to 0.853	0.262	−0.136 to 0.736	0.515	−0.561 to 0.893
Foot angle	0.977	0.928-0.995	0.992	0.975-0.998	0.995	0.984-0.999	0.998	0.995-1.000
Stance phase duration	0.917	0.762-0.981	0.971	0.906-0.994	0.925	0.781-0.983	0.974	0.915-0.994
Swing phase duration	0.944	0.834-0.987	0.981	0.938-0.996	0.953	0.857-0.989	0.984	0.947-0.996
%Stance phase duration	0.983	0.946-0.996	0.994	0.981-0.999	0.890	0.695-0.974	0.960	0.872-0.971
%Swing phase duration	0.983	0.946-0.996	0.994	0.981-0.999	0.890	0.695-0.974	0.960	0.872-0.971
Strike angle	0.964	0.890-0.992	0.988	0.961-0.997	0.895	0.707-0.976	0.962	0.879-0.992
Toe-off angle	0.971	0.912-0.994	0.990	0.969-0.998	0.953	0.858-0.989	0.984	0.948-0.996
Landing impact	0.940	0.822-0.986	0.979	0.933-0.995	0.940	0.823-0.986	0.979	0.933-0.995
Pronation	0.909	0.741-0.979	0.968	0.896-0.993	0.979	0.934-0.995	0.993	0.977-0.998
Lateral maximum displacement	0.655	0.264-0.907	0.851	0.519-0.967	0.716	0.355-0.927	0.883	0.623-0.974
Lateral minimum displacement	0.819	0.541-0.956	0.932	0.780-0.985	0.649	0.257-0.906	0.847	0.509-0.966
Vertical height	0.847	0.599-0.963	0.943	0.818-0.988	0.945	0.836-0.988	0.981	0.939-0.996

CI: confidence interval; CV: coefficient of variation; ICC: intraclass correlation coefficient.

### Inter-rater reliability

#### Differences by distance

The ICC_(2,1)_ values for stride length, foot angle, %stance phase duration, %swing phase duration, landing impact, and pronation showed excellent agreement (ICC_(2,1)_ ≥0.9). Speed, swing phase duration, strike angle, toe-off angle, lateral minimum displacement, and vertical height showed good agreement (ICC_(2,1)_ ≥0.75). Cadence, stride duration, stance phase duration, and lateral maximum displacement showed moderate agreement (ICC_(2,1)_: 0.5-0.75). The stride CV showed poor agreement, with an ICC_(2,1)_ of <0.5 (Table 4).

**Table 4.** Inter-Rater Reliability of the Motion Sensor-Based Gait Analysis System for Two Different Walking Distances and Examiners.

	Relative reliability		Absolute reliability					Random error			
	ICC_(2,1)_	95% CI	Fixed bias 95% CI for the mean difference	LOA (lower limit, upper limit)	Proportional bias β (95% CI)	P	Systemic bias	SEM	MDC	%SEM	%MDC
**Differences by distance (10 vs. 30 m)**											
Speed	0.839	0.464-0.961	-0.02 to 0.11	-0.13, 0.22	-0.014 (-0.513 to 0.485)	0.950	N	0.06	0.17	5.27	14.60
Cadence	0.673	0.131-0.913	-0.68 to 3.46	-3.89, 6.67	-0.580 (-1.116 to -0.044)	0.038	Y	1.90	5.28	3.30	9.15
Stride duration	0.593	0.015-0.887	-0.07 to 0.01	-0.14, 0.08	-0.685 (-1.283 to 0.087)	0.030	Y	0.04	0.11	3.79	10.50
Stride length	0.911	0.671-0.979	-0.04 to 0.07	-0.12, 0.15	0.062 (-0.331 to 0.455)	0.719	N	0.05	0.14	4.06	11.27
Stride CV	-0.691	-1.083 to 0.079	-1.28 to 0.83	-2.91, 2.47	-0.150 (-3.705 to 3.406)	0.924	N	0.97	2.69	35.23	97.64
Foot angle	0.996	0.981-0.999	-0.74 to 0.78	-1.92, 1.96	-0.014 (-0.102 to 0.073)	0.713	N	0.70	1.94	4.50	12.47
Stance phase duration	0.533	-0.070 to 0.867	-0.05 to 0.01	-0.10, 0.06	-0.328 (-1.233 to 0.577)	0.420	N	0.03	0.08	4.14	11.48
Swing phase duration	0.854	0.496-0.965	-0.02 to 0.00	-0.05, 0.03	-0.293 (-0.677 to 0.090)	0.113	N	0.01	0.04	3.65	10.12
%Stance phase duration	0.939	0.753-0.986	-0.70 to 0.61	-1.72, 1.63	1.056 (0.988-1.123)	<0.001	Y	0.61	1.68	0.93	2.57
%Swing phase duration	0.939	0.753-0.986	-0.61 to 0.70	-1.63, 1.72	1.056 (0.988-1.123)	<0.001	Y	0.61	1.68	1.74	4.83
Strike angle	0.892	0.616-0.974	-1.88 to 0.89	-4.02, 3.03	0.281 (-0.074 to 0.636)	0.103	N	1.27	3.53	8.04	22.29
Toe-off angle	0.883	0.288-0.976	-4.02 to -0.55	-6.71, 2.14	0.061 (-0.260 to 0.383)	0.665	Y	1.60	4.42	2.84	7.87
Landing impact	0.958	0.837-0.990	-2.29 to 4.80	-7.80, 10.31	-0.162 (-0.385 to 0.060)	0.129	N	3.27	9.05	4.86	13.46
Pronation	0.985	0.936-0.997	-0.80 to 0.81	-2.05, 2.06	-0.033 (-0.194 to 0.128)	0.646	N	0.74	2.06	9.50	26.31
Lateral maximum displacement	0.625	-0.038 to 0.902	-0.41 to 0.60	-1.19, 1.39	-0.316 (-1.159 to 0.527)	0.405	N	0.47	1.29	28.23	78.24
Lateral minimum displacement	0.845	0.443-0.963	-0.21 to 0.19	-0.52, 0.50	0.340 (-0.114 to 0.795)	0.120	N	0.18	0.51	20.67	57.29
Vertical height	0.873	0.556-0.969	-0.33 to 0.68	-1.12, 1.46	0.132 (-0.329 to 0.593)	0.520	N	0.47	1.29	2.78	7.70
**Differences by examiner (A vs. B)**											
Speed	0.716	0.177-0.927	-0.12 to 0.06	-0.26, 0.20	0.088 (-0.645 to 0.821)	0.785	N	0.08	0.23	7.07	19.59
Cadence	0.451	-0.267 to 0.844	-3.89 to 2.04	-8.48, 6.63	0.061 (-1.057 to 1.178)	0.901	N	2.73	7.55	4.75	13.17
Stride duration	0.365	-0.375 to 0.812	-0.04 to 0.08	-0.13, 0.17	0.225 (-0.997 to 1.448)	0.676	N	0.05	0.15	5.15	14.26
Foot angle	0.575	-0.149 to 0.888	-5.81 to 6.02	-14.97, 15.18	0.740 (0.033-1.447)	0.043	Y	5.44	15.08	34.62	95.96
Stride length	0.860	0.513-0.966	-0.08 to 0.05	-0.18, 0.14	-0.109 (-0.605 to 0.387)	0.618	N	0.06	0.16	4.73	13.12
Stride CV	0.335	-0.451 to 0.805	-1.10 to 0.78	-2.55, 2.24	-0.352 (-1.605 to 0.902)	0.528	N	0.86	2.39	32.04	88.82
Stance phase duration	0.334	-0.419 to 0.802	-0.03 to 0.05	-0.09, 0.11	0.040 (-1.244 to 1.324)	0.944	N	0.04	0.10	5.36	14.86
Swing phase duration	0.700	0.135-0.923	-0.01 to 0.03	-0.05, 0.06	0.144 (-0.612 to 0.899)	0.666	N	0.02	0.05	5.29	14.65
%Stance phase duration	0.879	0.548-0.972	-0.85 to 0.76	-2.10, 2.01	0.967 (0.851-1.083)	<0.001	Y	0.74	2.06	1.14	3.15
%Swing phase duration	0.879	0.548-0.972	-0.76 to 0.85	-2.01, 2.10	0.967 (0.851-1.083)	<0.001	Y	0.74	2.06	2.13	5.91
Strike angle	0.795	0.354-0.949	-0.59 to 3.30	-3.60, 6.30	-0.297 (-0.793 to 0.198)	0.199	N	1.79	4.95	10.90	30.22
Toe-off angle	0.831	0.430-0.959	-2.15 to 3.68	-6.66, 8.20	-0.214 (-0.741 to 0.312)	0.368	N	2.68	7.43	4.79	13.28
Landing impact	0.675	0.036-0.918	-10.04 to 8.71	-24.58, 23.25	0.305 (-0.472 to 1.082)	0.384	N	8.63	23.91	13.37	37.05
Pronation	0.545	-0.165 to 0.877	-2.82 to 4.42	-8.44, 10.04	0.724 (-0.039 to 1.487)	0.060	N	3.33	9.24	38.53	106.81
Lateral maximum displacement	0.780	0.279-0.946	-0.40 to 0.54	-1.13, 1.27	-0.221 (-0.847 to 0.405)	0.431	N	0.43	1.20	25.88	71.73
Lateral minimum displacement	0.460	-0.314 to 0.851	-0.30 to 0.36	-0.82, 0.88	-0.180 (-1.293 to 0.933)	0.714	N	0.31	0.85	35.51	98.43
Vertical height	0.668	0.052-0.915	-0.94 to 0.58	-2.11, 1.75	-0.094 (-0.913 to 0.725)	0.793	N	0.70	1.93	4.19	11.61

β: partial regression coefficient; CI: confidence interval; CV: coefficient of variation; ICC: intraclass correlation coefficient; LOA: limits of the agreement; MDC: minimal detectable change; N: no; SEM: standard error of measurement; Y: yes.

#### Differences by examiner

No gait parameters reached excellent reliability (ICC_(2,1)_ ≥0.9). Stride length, %stance phase duration, %swing phase duration, strike angle, toe-off angle, and lateral maximum displacement showed good agreement (ICC_(2,1)_ ≥0.75). Speed, foot angle, swing phase duration, landing impact, pronation, and vertical height showed moderate agreement (ICC_(2,1)_: 0.5-0.75). Cadence, stride duration, stride CV, stance phase duration, and lateral minimum displacement showed poor agreement (ICC_(2,1)_ <0.5) (Table 4).

#### Differences by timing

Stride length, swing phase duration, %stance phase duration, and %swing phase duration showed excellent agreement for both examiners A and B (ICC_(2,1)_ ≥0.9). Speed, strike angle, toe-off angle, landing impact, and maximum vertical height showed good agreement (ICC_(2,1)_ ≥0.75). Reliability for other parameters varied between examiners A and B (Table 5).

**Table 5.** Inter-Rater Reliability of the Motion Sensor-Based Gait Analysis System for Two Timings as Evaluated by Each of the Two Examiners.

	Relative reliability		Absolute reliability					Random error			
	ICC_(2,1)_	95% CI	Fixed bias 95% CI for the mean difference	LOA (lower limit, upper limit)	Proportional bias β (95% CI)	P	Systemic bias	SEM	MDC	%SEM	%MDC
**Examiner A**											
Speed	0.843	0.442-0.962	-0.11 to 0.01	-0.20, 0.11	0.106 (-0.353 to 0.566)	0.601	N	0.06	0.16	4.80	13.30
Cadence	0.649	0.093-0.906	-3.36 to 0.78	-6.58, 3.99	-0.158 (-0.934 to 0.619)	0.646	N	1.91	5.28	3.33	9.24
Stride duration	0.603	0.020-0.891	-0.02 to 0.06	-0.08, 0.12	-0.085 (-0.943 to 0.773)	0.821	N	0.04	0.10	3.48	9.65
Stride length	0.952	0.796-0.989	-0.06 to 0.01	-0.12, 0.06	0.022 (-0.235 to 0.279)	0.846	N	0.03	0.09	2.64	7.33
Stride CV	0.588	-0.028 to 0.888	-0.44 to 1.17	-1.68, 2.41	-0.010 (-0.916 to 0.897)	0.981	N	0.74	2.04	24.92	69.08
Foot angle	0.931	0.736-0.984	-1.09 to 2.00	-3.49, 4.40	-0.013 (-0.362 to 0.336)	0.932	N	1.42	3.95	9.16	25.40
Stance phase duration	0.492	-0.128 to 0.852	-0.01 to 0.05	-0.06, 0.10	-0.222 (-1.218 to 0.774)	0.614	N	0.03	0.08	4.12	11.43
Swing phase duration	0.915	0.691-0.980	-0.01 to 0.02	-0.02, 0.03	0.118 (-0.236 to 0.473)	0.455	N	0.01	0.03	2.71	7.51
%Stance phase duration	0.922	0.713-0.982	-0.42 to 0.95	-1.49, 2.01	0.080 (-0.277 to 0.437)	0.613	N	0.63	1.75	0.97	2.68
%Swing phase duration	0.922	0.713-0.982	-0.95 to 0.42	-2.01, 1.49	0.080 (-0.277 to 0.437)	0.613	N	0.63	1.75	1.82	5.05
Strike angle	0.944	0.778-0.987	-1.41 to 1.00	-3.27, 2.86	-0.129 (-0.424 to 0.167)	0.337	N	1.11	3.06	6.44	17.85
Toe-off angle	0.767	0.265-0.942	-2.78 to 4.64	-8.54, 10.40	-0.018 (-0.688 to 0.653)	0.952	N	3.41	9.47	6.11	16.94
Landing impact	0.792	0.359-0.948	-10.81 to 2.72	-21.29, 13.20	0.231 (-0.315 to 0.777)	0.351	N	6.22	17.24	9.90	27.44
Pronation	0.940	0.756-0.986	-0.91 to 0.88	-2.30, 2.26	-0.036 (-0.367 to 0.295)	0.803	N	0.82	2.28	9.08	25.16
Lateral maximum displacement	0.913	0.683-0.979	-0.19 to 0.41	-0.65, 0.88	-0.113 (-0.486 to 0.261)	0.499	N	0.28	0.77	16.27	45.09
Lateral minimum displacement	0.839	0.465-0.961	-0.29 to 0.13	-0.61, 0.46	0.190 (-0.319 to 0.699)	0.406	N	0.19	0.54	21.07	58.41
Vertical height	0.841	0.443-0.962	-0.66 to 0.47	-1.53, 1.34	0.033 (-0.515 to 0.582)	0.890	N	0.52	1.43	3.09	8.58
**Examiner B**											
Speed	0.921	0.702-0.982	-0.04 to 0.07	-0.12, 0.15	0.079 (-0.289 to 0.447)	0.628	N	0.05	0.13	4.13	11.46
Cadence	0.927	0.731-0.983	-1.68 to 0.73	-3.55, 2.59	0.178 (-0.133 to 0.490)	0.218	N	1.11	3.07	1.95	5.42
Stride duration	0.922	0.713-0.981	-0.01 to 0.04	-0.05, 0.07	0.195 (-0.121 to 0.510)	0.188	N	0.02	0.06	2.10	5.81
Stride length	0.906	0.662-0.978	-0.02 to 0.08	-0.10, 0.15	0.128 (-0.242 to 0.497)	0.440	N	0.05	0.13	3.71	10.28
Stride CV	0.454	-0.214 to 0.842	-0.41 to 1.07	-1.56, 2.22	0.025 (-1.068 to 1.118)	0.959	N	0.68	1.89	24.48	67.85
Foot angle	0.650	0.040-0.909	-6.62 to 3.41	-14.39, 11.19	-0.905 (-1.130 to -0.680)	<0.001	Y	4.61	12.79	28.03	77.69
Stance phase duration	0.878	0.582-0.971	-0.01 to 0.03	-0.04, 0.06	0.182 (-0.238 to 0.601)	0.340	N	0.02	0.05	2.46	6.82
Swing phase duration	0.960	0.839-0.991	-0.01 to 0.01	-0.02, 0.03	0.202 (0.008-0.397)	0.043	Y	0.01	0.02	2.30	6.39
%Stance phase duration	0.928	0.730-0.983	-0.44 to 0.83	-1.43, 1.81	0.209 (-0.092 to 0.510)	0.144	N	0.58	1.62	0.89	2.48
%Swing phase duration	0.928	0.730-0.983	-0.83 to 0.44	-1.81, 1.43	0.209 (-0.092 to 0.510)	0.144	N	0.58	1.62	1.68	4.66
Strike angle	0.823	0.414-0.957	-2.83 to 0.47	-5.38, 3.02	0.151 (-0.345 to 0.647)	0.495	N	1.52	4.20	9.30	25.77
Toe-off angle	0.924	0.710-0.982	-1.41 to 2.19	-4.19, 4.97	0.035 (-0.322 to 0.403)	0.826	N	1.65	4.58	2.99	8.28
Landing impact	0.898	0.613-0.976	-5.00 to 6.55	-13.96, 15.51	-0.059 (-0.492 to 0.373)	0.755	N	5.31	14.73	8.23	22.81
Pronation	0.687	0.152-0.918	-4.45 to 1.26	-8.88, 5.68	-0.657 (-1.119 to -0.194)	0.012	Y	2.63	7.28	29.03	80.46
Lateral maximum displacement	0.575	-0.033 to 0.882	-0.26 to 0.82	-1.11, 1.67	-0.069 (-0.980 to 0.843)	0.864	N	0.50	1.39	27.04	74.94
Lateral minimum displacement	0.412	-0.257 to 0.825	-0.15 to 0.41	-0.59, 0.85	-0.365 (-1.467 to 0.736)	0.459	N	0.26	0.72	33.10	91.76
Vertical height	0.906	0.662-0.978	-0.17 to 0.62	-0.79, 1.24	0.166 (-0.187 to 0.518)	0.303	N	0.36	1.01	2.19	6.06

β: partial regression coefficient; CI: confidence interval; CV: coefficient of variation; ICC: intraclass correlation coefficient; LOA: limits of the agreement; MDC: minimal detectable change; N: no; SEM: standard error of measurement; Y: yes.

### Absolute reliability assessment

#### Systematic bias

**Figure 3.**
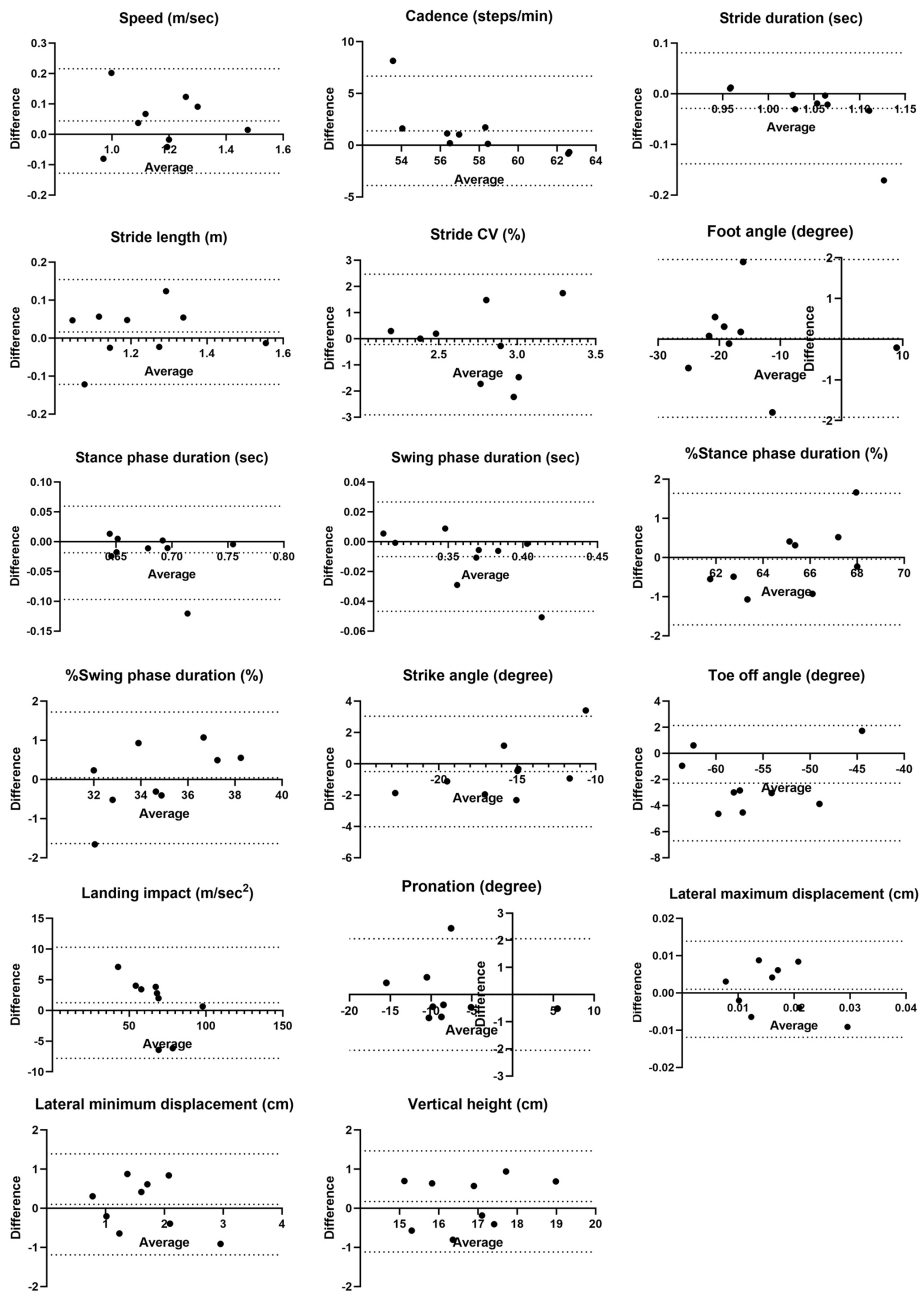
Bland-Altman plots of the average and difference of gait parameters for 10-m and 30-m walking. The dotted line represents the mean difference between the two measurements and the 95% confidence interval.

Fixed bias was observed in toe-off angle regarding distance differences. Proportional biases were observed in cadence, stride duration, %stance phase duration, and %swing phase duration, with one of the nine cases exceeding the LOA in the Bland-Altman analysis (Figure 3).

**Figure 4.**
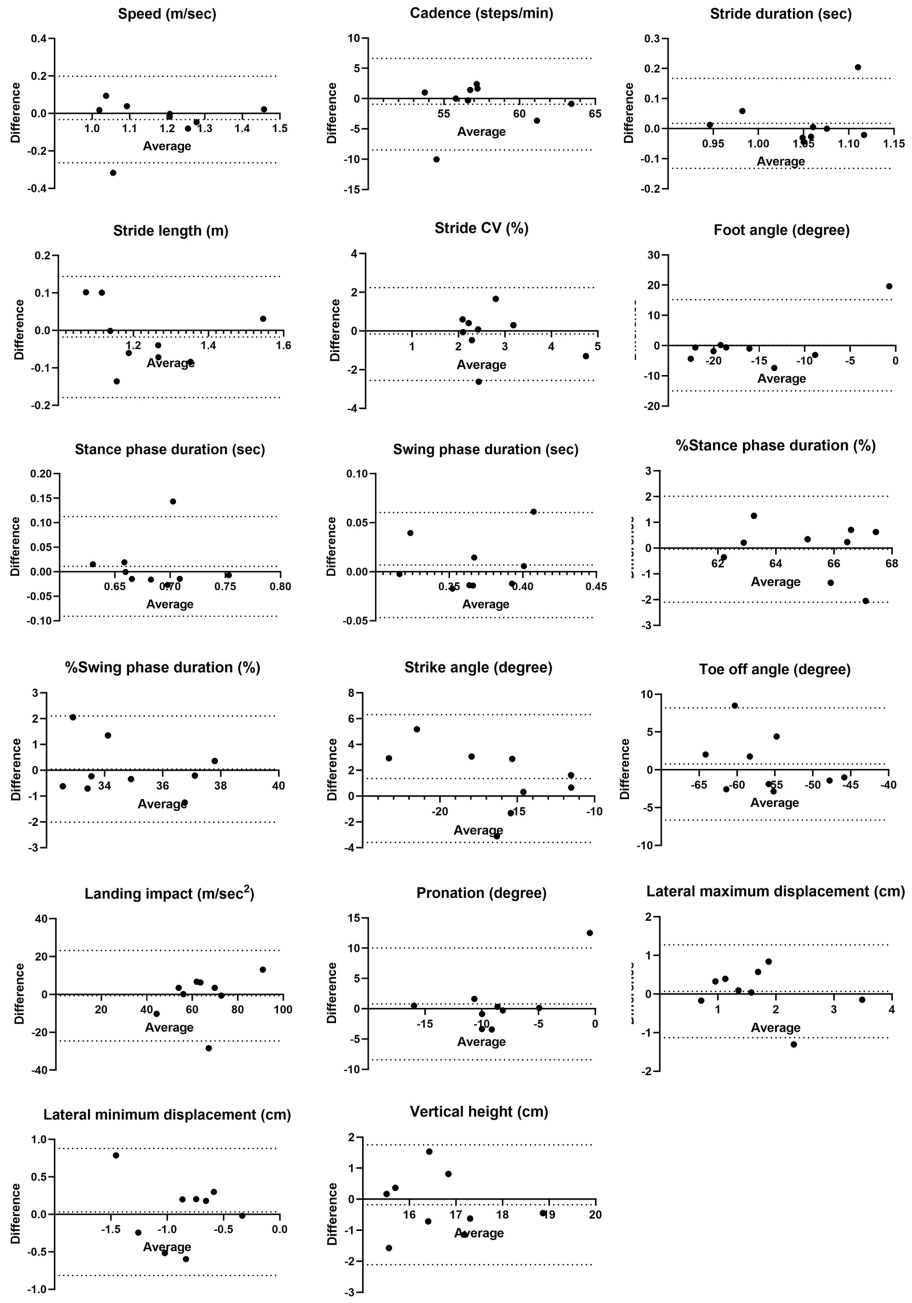
Bland-Altman plots of the average and difference of gait parameters assessed by examiners A and B. The dotted line represents the mean difference between the two measurements and the 95% confidence interval.

No fixed bias was observed between examiners, but proportional biases were found in foot angle, %stance phase duration, and %swing phase duration. Foot angle exceeded the LOA in one out of nine cases, while %stance and %swing phase duration were near the LOA limits in one case each (Figure 4).

**Figure 5.**
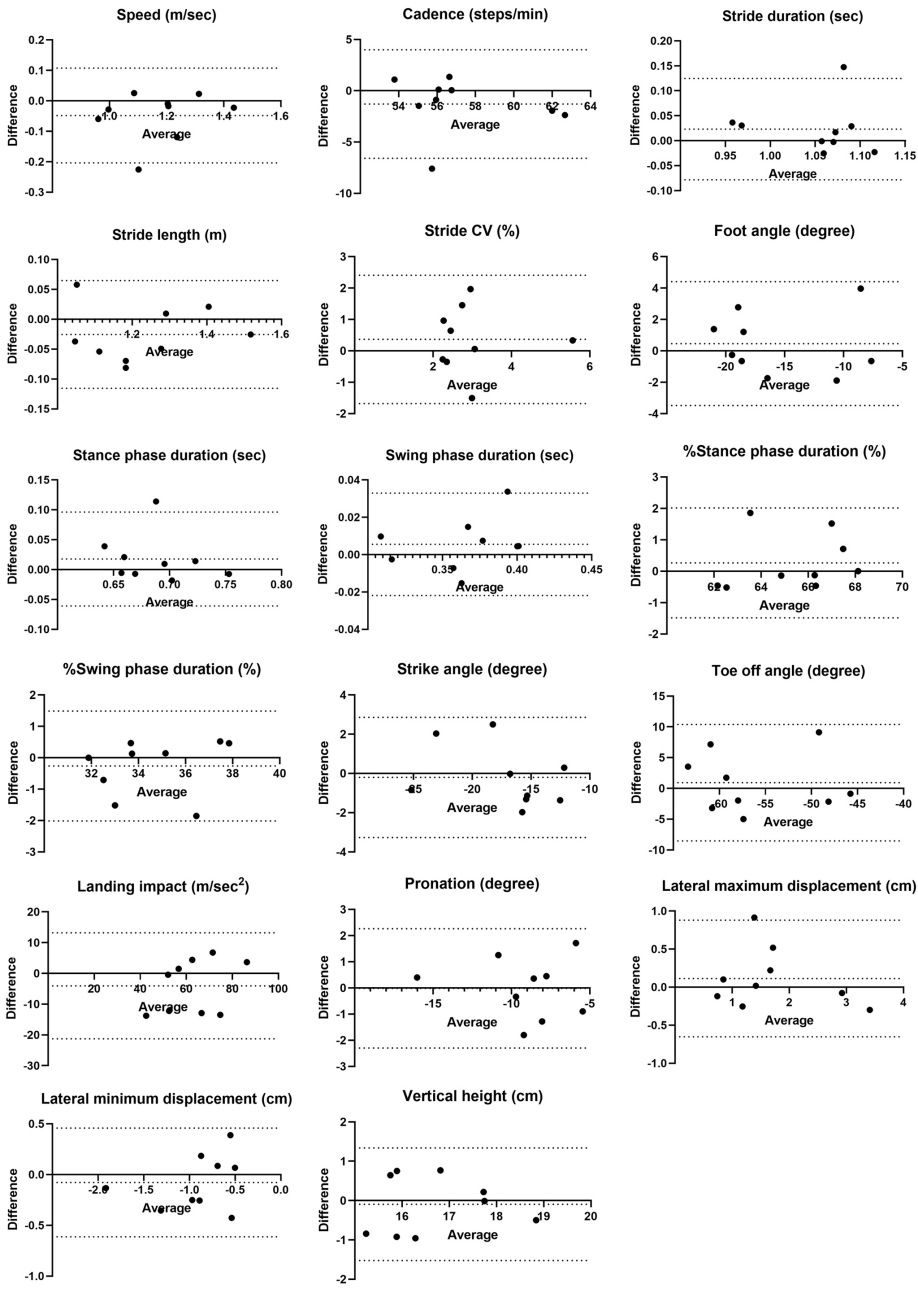
Bland-Altman plots of the average and difference of gait parameters in the morning and afternoon evaluated by examiner A. The dotted line represents the mean difference between the two measurements and the 95% confidence interval.

**Figure 6.**
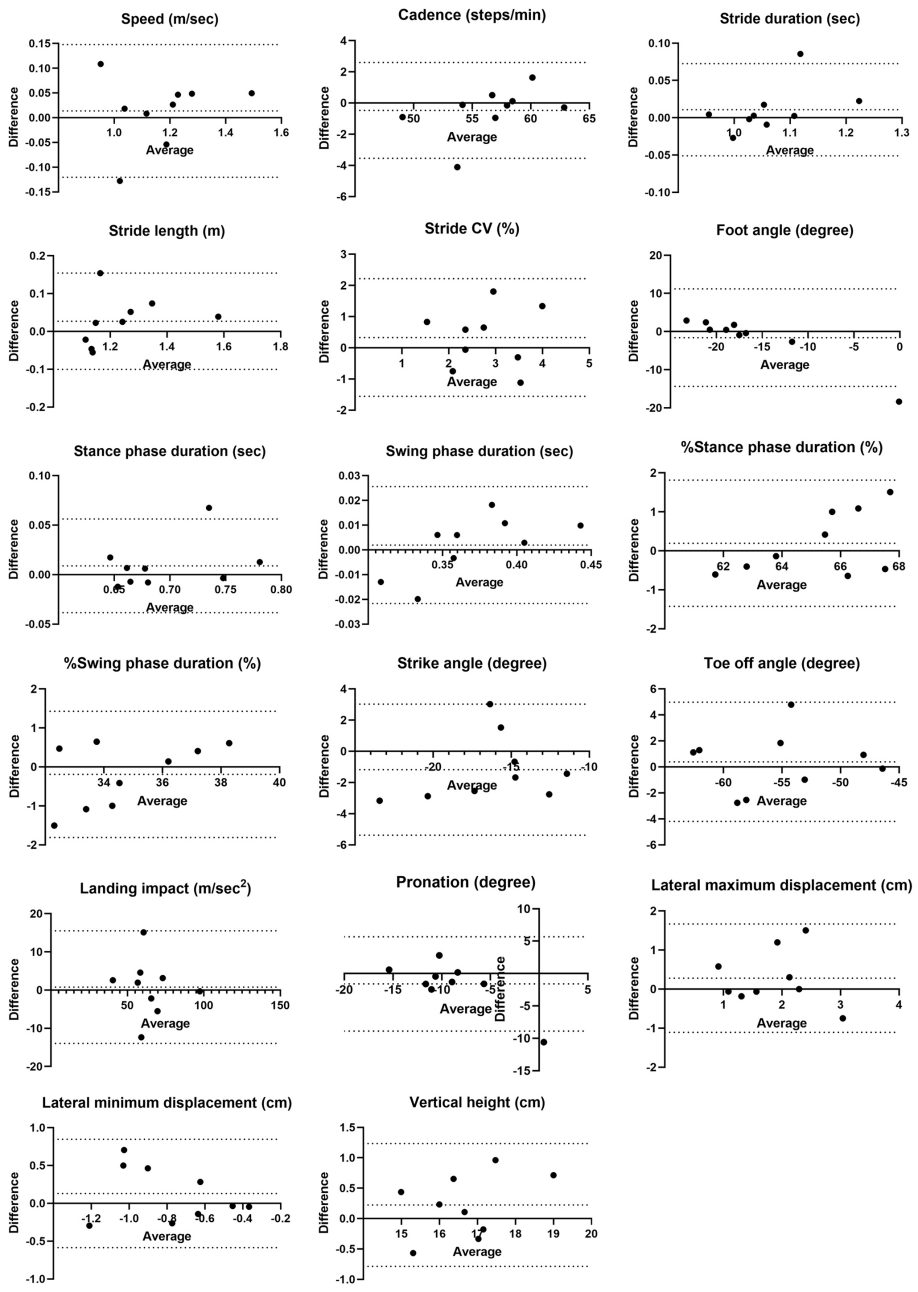
Bland-Altman plots of the average and difference of gait parameters in the morning and afternoon evaluated by examiner B. The dotted line represents the mean difference between the two measurements and the 95% confidence interval.

Examiner A showed no systematic bias related to time differences (Figure 5), but examiner B exhibited proportional biases in foot angle and swing phase duration (Figure 6). For examiner B, one case exceeded the lower limit of the LOA for foot angle and another for swing phase duration near the lower limit of the LOA.

#### Random error

For distance differences, stride CV, lateral maximum displacement, and lateral minimum displacement had %MDC values ≥40%, indicating existence of substantial random error. Similarly, %MDC values ≥40% were observed for foot angle, stride CV, pronation, and lateral minimum displacement across the examiners. Both examiners showed large %MDC errors for stride CV, lateral maximum displacement, and lateral minimum displacement. Examiner B additionally showed %MDC values ≥40% for foot angle and pronation.

## Discussion

This study found three key points to clarify the reliability of ORPHE ANALYTICS, a gait analysis motion sensor system, in patients with T2D. First, the intra-rater reliability was good to excellent for all parameters except stride CV, with ICC_(1,1)_ values ranging from 0.87 to 0.97. Second, inter-rater reliability for differences in distance and timing was almost good, while inter-rater reliability between examiners was moderate to good, although stride CV consistently showed poor reliability. Third, regarding absolute reliability, the %MDC values were high for the lateral displacement during the swing phase and stride CV. Additionally, the %MDC values exceeded 40% for foot angle and pronation when examiners and timing differed.

### Relative reliability for intra-rater reliability in T2D

ORPHE ANALYTICS showed good to excellent reliability, with ICC_(1,1)_ values ≥0.75 for both examiners, except for stride CV. For comparison, the ICC_(1,1)_ values for speed and stride duration using other gait systems, such as Physilog (BioAGM, La Tour-de-Peilz, Switzerland) and GAITRite (CIR Systems, Franklin, NJ, USA) for individuals aged ≥70 years ranged from 0.82 to 0.95 and 0.85 to 0.90, respectively ^(23)^. While differences in participant characteristics should be considered, ORPHE ANALYTICS showed sufficient intra-rater reliability with ICC_(1,1)_ values of 0.963-0.975 for speed and 0.911-0.960 for stride duration. According to the Spearman-Brown formula, when ICC_(1,1)_ is 0.9 or more, a single measurement is sufficient to ensure excellent reliability ^(24)^. In our study, the intra-rater reliability of ORPHE ANALYTICS was found to be comparable to that of existing gait analysis systems. Physilog is a system that measures gait by attaching wearable motion sensors to both thighs and lower legs (fixed by elastic band) ^(23)^, while GAITRite is a system that captures gait data by walking on a mat embedded with sensors ^(23), (25)^. The ORPHE CORE used in this study was embedded in the midsole, which may have made its position more stable than that of the Physilog fixation by elastic band. Additionally, unlike GAITRite, ORPHE CORE has no restrictions on measurement distance, allowing for gait analysis under more stable walking conditions by ensuring a sufficient walking distance ^(12), (13), (26)^. Although not statistically significant, the higher ICC_(1,1)_ for a 20-m walking distance compared with a 5-m distance in the Physilog intra-rater reliability report (0.95 [95% CI 0.89-0.98] vs. 0.82 [95% CI 0.63-0.92]) ^(23)^ suggests that longer walking distances contribute to more stable and reliable measurements. This further supports the reliability of ORPHE ANALYTICS.

### Relative reliability and absolute reliability for inter-rater reliability in T2D

The inter-rater reliability for differences in distance and timing was considered good to excellent, except for some parameters. Some gait parameters showing high reliability (speed or stride length) were associated with falls ^(27), (28), (29)^ and cognitive function ^(30), (31)^ in patients with diabetes. These findings support the clinical applicability of ORPHE ANALYTICS. However, cadence, stride duration, and stride CV had inconsistent ICC_(2,1)_ values under different conditions. Previous studies using GAITRite reported improved reliability for cadence and duration when the number of assessed steps increased ^(13)^, and stride CV required even more steps for better reliability ^(12), (13)^. Our results were consistent with these results. Although the systematic biases in cadence and stride duration were likely influenced by a single outlier (Figure 3), the LOAs (lower limit, upper limit) for cadence (−3.89, 6.67) and stride duration (−0.14, 0.08) in the population were within acceptable ranges compared with previously reported values for patients with T2D: 110.71 (9.11) steps/min and 1.22 (0.04) seconds ^(9), (27)^, respectively.

Inter-rater reliability between examiners was fair to moderate for most parameters, except for lateral maximum displacement, stride, toe-off angle, and stance (swing) phase percentage. To the best of our knowledge, this is the first report evaluating the inter-rater reliability of ORPHE ANALYTICS in patients with diabetes, albeit with some unresolved issues. Unlike the results from FeetMe (FeetMe, Paris, France), which reported ICC_(2,1)_ values >0.9 for speed and cadence in a population without diabetes ^(32)^, our results were obtained from assessments conducted by multiple examiners on the same day. ORPHE ANALYTICS showed lower reliability for speed (ICC_(2,1)_: 0.716) and cadence (ICC_(2,1)_: 0.451). However, in our study, it should be noted that inter-rater reliability was assessed under conditions that also incorporated an aspect of test-retest reliability, as assessments were conducted on different days. Therefore, the observed variability may not solely reflect differences between examiners but also fluctuations in gait parameters over time. In a previous study using accelerometers to evaluate test-retest reliability in patients with T2D, with assessments conducted by the same examiner at weekly intervals, the ICC_(2,1)_ for walking speed and cadence was reported as 0.824 and 0.834, respectively ^(33)^. That study also found significantly greater stride variability in patients with neuropathy. In our study, 88.9% of the participants had neuropathy, which may have contributed to variations in stride length, leading to fluctuations in speed and reduced reliability. Overall, the inter-rater reliability in our study may reflect not only differences between examiners but also changes in participants’ gait parameters due to differences in the timing of the assessments. To exclude variability of gait parameters in study participants and accurately assess true inter-rater reliability, future studies may need to evaluate participants using different examiners on the same day at the same time.

The LOAs for speed (−0.26, 0.20), cadence (−8.48, 6.63), and stride duration (−0.13, 0.17) in the sample population were relatively wide. However, the estimated LOAs for the most optimistic case in the population were (−0.11, 0.04), (−3.35, 1.50), and (−0.03, 0.07), which fell within the acceptable range when compared with reported ranges in patients with T2D (1.21 [0.18] m/sec for speed, along with corresponding cadence and duration values) ^(9)^. However, the position paper on gait analysis by the Italian Society of Clinical Movement Analysis (SIAMOC) recommends using the same examiner to improve measurement reliability ^(34)^. Our results with ORPHE ANALYTICS align with this recommendation.

Inter-rater reliability between the morning and afternoon sessions differed between examiners A and B, with some gait parameters having different ICCs. Conversely, the stride CV was fair to moderate for both examiners, with ICC_(2,1)_ of 0.588 and 0.454, respectively. The stride CV for our entire study population averaged 2.70%-3.12% (Table 2), which was similar to a study using motion sensors on patients with diabetes (reported average: 2.0%-4.5%); thus, the insufficient reliability was not influenced by participant heterogeneity ^(35)^. The gait analysis by ORPHE ANALYTICS was limited to data within the quartile range of each gait parameter’s distribution, since we used the software results without modification to explore clinical application potential. This analytical approach may lead to inaccurate results when analyzing gait parameters that depend on the number of steps used in the analysis, such as stride duration CV, particularly when the measurement distance is short. Furthermore, the poor reliability expressed by the ICC for stride CV was due to the large variability in individual measurements, since the %MDC exceeds 40% in all conditions. This is also suggested by the relatively low intra-rater reliability (ICC_(1,1)_, ICC_(1,3)_) for stride CV, as patients with diabetes have greater variability in step length than healthy people ^(27), (36), (37)^. Stride length variability in patients with diabetes and older adults is associated with age ^(38)^, diabetic neuropathy ^(39)^, and lower-limb muscle strength ^(40)^. Furthermore, gait variability in diabetes and older adults has been linked to falls ^(41)^, frailty ^(42)^, and dementia ^(43)^. Future studies should investigate cross-sectional and longitudinal relationships between high gait variability and comorbidities in patients with diabetes to clarify the clinical significance of high gait variability in these patients.

Our study revealed that lateral displacement during the swing phase, foot angle, and pronation had large %MDC (>40%) in certain conditions. What these three gait parameters have in common is that they involve movements in the mediolateral direction relative to the walking direction. This increased fluctuation in the mediolateral direction is observed in patients with diabetes ^(44)^ and is associated with age ^(45)^. The mechanism of this transition is not well understood, but it has been reported that changes in sensory systems and balance ability may affect variability in the medial and lateral directions ^(46)^. These highly variable gait parameters may also reflect lower-limb dysfunctions, such as ankle and knee impairments, commonly seen in patients with diabetes ^(47), (48)^. Furthermore, it has also been reported that foot pronation affects ankle and knee joint movement ^(49)^. Although these lower limb functional disorders may be related to gait variability, the causal relationship between gait changes and lower limb functional disorders remains unclarified ^(27)^. Further research is required to understand the relationship between these gait abnormalities and underlying conditions.

### Strengths and Limitations

This study has two strengths. Firstly, we evaluated both relative reliability (using ICC) and absolute reliability (using LOA and MDC based on Bland-Altman analysis) to assess clinical applicability. Second, we evaluated a comprehensive range of gait parameters beyond those typically assessed (e.g., speed, cadence, and stance/swing phase duration).

However, the present study has three limitations. First, the small sample size, due to the inclusion criterion requiring stable gait, limited generalizability, and larger studies evaluating reliability are needed in the future. Second, the inclusion of inpatients may also limit generalizability, as their activity levels and comorbidities differ from those of outpatients. Third, with a diabetic neuropathy rate of 88.9%, the impact on gait and variability of gait parameters could have influenced the results. Additionally, the system’s reliability in non-diabetic individuals was not assessed, precluding any controlled comparisons.

### Conclusions

In conclusion, the ORPHE ANALYTICS system demonstrates good to excellent intra-rater and inter-rater reliability for gait parameters based on differences in distance and timing, with acceptable absolute reliability. However, consistent examiner involvement is recommended to enhance reliability, since inter-rater reliability across different examiners was only moderate. New gait parameters exhibiting large variability, such as lateral displacement, foot angle, and pronation, were identified. These findings provide an important basis for the practical application of gait analysis in patients with diabetes and for improving devices.

## Article Information

### Conflicts of Interest

None

### Acknowledgement

We thank all study participants for their cooperation.

### Author Contributions

Takaaki Matsuda: Conceptualization, Data curation, Formal analysis, Investigation, Methodology, Project administration, Supervision, Visualization, Writing - original draft, Writing - review and editing. Hirofumi Takahashi: Conceptualization, Investigation, Methodology, Writing - review and editing. Yoshinori Osaki, Erika Matsuda, Yuki Murayama, Yoko Sugano, Hitoshi Iwasaki, Motohiro Sekiya, Bryan J. Mathis: Supervision, Writing - review and editing. Yasuhiro Suzuki: Conceptualization, Supervision, Writing - review and editing. Kosuke Kojo: Formal analysis, Writing - review and editing. Hiroaki Suzuki: Funding acquisition, Methodology, Supervision, Writing - review and editing. Hitoshi Shimano: Project administration, Supervision, Writing - review and editing.

### Approval by Institutional Review Board (IRB)

This study was approved by the Ethics Committee of the University of Tsukuba Hospital (approval date: 27 July 2023, approval number: R05-058).

### Data Availability

Data will be made available on request.

## Supplement

Supplementary Table
