# Supplementary material for: Reliability of Gait Analysis Using ORPHE ANALYTICS in Patients with Type 2 Diabetes: A Single-center Observational Study
Source: JMA J. 2025 Jun 13;8(3):853–70. doi: 10.31662/jmaj.2024-0422 (PMC12328463; doi:10.31662/jmaj.2024-0422)
Supplement: Supplementary Table [file 2433-3298-8-3-0853-s001.pdf]

Supplementary Table 1. Estimated limit of agreement (LOA) in the population.

	95% CI for lower LOA	95% CI for upper LOA
**Differences by distance (10 m vs. 30m)**		
Speed	−0.25 to −0.01	0.10-0.33
Cadence	−7.48 to −0.31	3.08-10.25
Stride duration	−0.21 to −0.06	0.01-0.16
Stride length	−0.22 to −0.03	0.06-0.25
Stride CV	−4.74 to −1.08	0.64-4.30
Foot angle	−3.23 to −0.60	0.64-3.27
Stance phase duration	−0.15 to −0.04	0.01-0.11
Swing phase duration	−0.07 to −0.02	0.00-0.05
%Stance phase duration	−2.86 to −0.58	0.49-2.77
%Swing phase duration	−2.77 to −0.49	0.58-2.86
Strike angle	−6.42 to −1.62	0.64-5.43
Toe off angle	−9.71 to −3.70	−0.87 to 5.14
Landing impact	−13.94 to −1.65	4.16-16.45
Pronation	−3.45 to −0.66	0.66-3.46
Lateral maximum displacement	−2.07 to −0.32	0.51-2.26
Lateral minimum displacement	−0.87 to −0.18	0.15-0.84
Vertical height	−2.00 to −0.24	0.59-2.34
**Differences by examiners (A vs. B)**		
Speed	−0.42 to −0.11	0.04-0.36
Cadence	−13.61 to −3.35	1.50-11.76
Stride duration	−0.23 to −0.03	0.07-0.27
Foot angle	−25.22 to −4.73	4.94-25.42
Stride length	−0.29 to −0.07	0.03-0.25
Stride CV	−4.18 to −0.93	0.61-3.86
Stance phase duration	−0.16 to −0.02	0.04-0.18
Swing phase duration	−0.08 to −0.01	0.02-0.10
%Stance phase duration	−3.50 to −0.70	0.62-3.41
%Swing phase duration	−3.41 to −0.62	0.70-3.50
Strike angle	−6.96 to −0.23	2.94-9.67
Toe off angle	−11.71 to −1.62	3.15-13.24
Landing impact	−40.82 to −8.33	7.01-39.49
Pronation	−14.71 to −2.16	3.76-16.31
Lateral maximum displacement	−1.95 to −0.32	0.46-2.09
Lateral minimum displacement	−1.39 to −0.24	0.30-1.46
Vertical height	−3.42 to −0.80	0.44-3.07
**Examiner A**		
Speed	−0.31 to −0.10	0.00-0.21
Cadence	−10.16 to −2.99	0.40-7.58
Stride duration	−0.15 to −0.01	0.06-0.19
Foot angle	−6.17 to −0.81	1.72-7.08
Stride length	−0.18 to −0.05	0.00-0.13
Stride CV	−3.07 to −0.29	1.02-3.80
Stance phase duration	−0.11 to −0.01	0.04-0.15
Swing phase duration	−0.04 to 0.00	0.01-0.05
%Stance phase duration	−2.67 to −0.30	0.83-3.20
%Swing phase duration	−3.20 to −0.83	0.30-2.67
Strike angle	−5.35 to −1.19	0.78-4.94
Toe off angle	−14.96 to −2.11	3.97-16.83
Landing impact	−33.00 to −9.57	1.49-24.91
Pronation	−3.84 to −0.75	0.71-3.81
Lateral maximum displacement	−1.17 to −0.13	0.36-1.40
Lateral minimum displacement	−0.98 to −0.25	0.09-0.82
Vertical height	−2.50 to −0.55	0.36-2.31

**Examiner B**			
	Speed	−0.21 to −0.03	0.06-0.24
	Cadence	−5.63 to −1.46	0.51-4.68
	Stride duration	−0.09 to −0.01	0.03-0.11
	Foot angle	−23.08 to −5.71	2.50-19.87
	Stride length	−0.19 to −0.01	0.07-0.24
	Stride CV	−2.84 to −0.28	0.93-3.50
	Stance phase duration	−0.07 to −0.01	0.02-0.09
	Swing phase duration	−0.04 to −0.01	0.01-0.04
	%Stance phase duration	−2.53 to −0.33	0.71-2.91
	%Swing phase duration	−2.91 to −0.71	0.33-2.53
	Strike angle	−8.23 to −2.53	0.17-5.87
	Toe off angle	−7.31 to −1.08	1.86-8.09
	Landing impact	−23.96 to −3.95	5.50-25.51
	Pronation	−13.82 to −3.93	0.74-10.63
	Lateral maximum displacement	−2.05 to −0.17	0.72-2.61
	Lateral minimum displacement	−1.07 to −0.10	0.36-1.33
	Vertical height	−1.47 to −0.10	0.55-1.92

CI: confidence interval, LOA: limits of the agreement, CV: coefficient of variation.
